# Improving pre-exposure prophylaxis uptake among Black women in the United States: insights from conducting public health research within emergency department setting

**DOI:** 10.3389/fsoc.2025.1613227

**Published:** 2025-08-15

**Authors:** Hema Sarvani Jalaparthi, Mandy J. Hill

**Affiliations:** ^1^Substance Use Research and Education (SURE) Center, College of Pharmacy, University of New Mexico Health Science Center, Albuquerque, NM, United States; ^2^Department of Population Health and Health Disparities, Centennial Chair in Preventive Medicine and Community Health, School of Public and Population Health, University of Texas Medical Branch (UTMB), Galveston, TX, United States

**Keywords:** PrEP uptake, HIV prevention, emergency department setting, Black women, public health research

## Introduction

The intersection of public health research and emergency medicine presents a valuable opportunity to address health disparities, particularly among populations disproportionately affected by the Human Immunodeficiency Virus (HIV). Despite significant progress toward ending the HIV epidemic (EHE) in some United States (U.S.), African American/Black women in the southern U.S. continue to experience more new HIV cases than other women and remain underrepresented in the utilization of preventive services such as pre-exposure prophylaxis (PrEP; Arnold et al., 2022). Structural barriers such as healthcare access, medical mistrust, and stigma contribute to these disparities, necessitating innovative approaches to improve awareness and uptake (Crooks et al., 2023). Emergency departments (EDs) serve as key access points to healthcare for many individuals; as such, the ED visit provides a unique opportunity to implement public health interventions and connect HIV-vulnerable individuals to preventive services (Shull et al., 2020). This opinion examines the intersectionality through the lens of a pilot project aimed at increasing knowledge and facilitating uptake of PrEP to prevent new incident HIV cases among Black women. This narrative highlights how integrating public health research strategies within ED settings can enhance preventive care and improve health outcomes for underserved populations.

## Persistent challenges with PrEP uptake among black women

Pre-exposure prophylaxis is a medication regimen taken to reduce the chance of becoming HIV positive (Arnold et al., 2022; NIH, 2021). It is available either as a once-daily pill: emtricitabine/tenofovir disoproxil fumarate (Truvada^®^) and emtricitabine/tenofovir alafenamide (Descovy^®^); or as a long-acting injectable option, including cabotegravir (Apretude^®^), administered every 2 months. More recently, lenacapavir (Yeztugo^®^), a long-acting injectable administered twice yearly, has also been approved, offering an alternative for individuals who face challenges with daily adherence or consistent access to healthcare (NIH, 2021; HIV, 2025; WHO, 2025). The Centers for Disease Control and Prevention (CDC) recommend that all sexually active adults and adolescents should be informed about PrEP (CDC, 2021). Although there is high access to and uptake of PrEP, many groups, particularly Black women, still face barriers to access and initiate PrEP (Gormley et al., 2023). Fewer than two percent of cisgender Black women indicated for PrEP in the U.S. have received a prescription (Willie and Dale, 2024). While many Black women are open to using PrEP for HIV prevention, systemic barriers continue to result in low awareness and uptake of PrEP (Smit and Masvawure, 2023; Troutman et al., 2021; Clement et al., 2024).

Using the ecological framework, we can understand the PrEP barriers at multiple levels. Individual-level barriers include lack of HIV risk awareness, concerns about adherence, potential side effects, effectiveness, challenges with maintaining a consistent medication schedule and awareness of PrEP (Arnold et al., 2022; Crooks et al., 2023). Interpersonal-level barriers include the influence of sexual and romantic partners, and stigma from family (Smit and Masvawure, 2023). Societal-level barriers encompass insufficient PrEP marketing that are inclusive of Black women, historical mistrust and/or distrust of the medical system, inadequate communication from healthcare providers, cost, and structural violence (Smit and Masvawure, 2023; Crooks et al., 2023; Nydegger and Hill, 2020; Lambert et al., 2018; Hill et al., 2025c).

Racial stigma is a significant structural barrier rooted in historical mistreatment and ongoing discrimination within healthcare systems, and it profoundly impacts PrEP uptake among Black communities (Crooks et al., 2023). Additionally, poverty and other social determinants of health (SDOH) further limit access to preventive services. People in poverty often have trouble getting to healthcare appointments due to lack of transportation, and managing appointments and follow-up care can be especially difficult for those balancing housing, employment and other responsibilities (Boudreaux et al., 2025; Sims Haynes et al., 2024). This framework underscores the need for a comprehensive, multilevel strategy that addresses the interconnected layers to improve PrEP access, utilization, and ultimately promote more equitable health outcomes.

## Enhancing PrEP outreach and healthcare provider engagement

To effectively enhance outreach and engage Black women with PrEP, education and counseling must be tailored to their cultural needs and preferences (Arnold et al., 2022). Providing resources or education in community settings where women regularly congregate, leveraging social media platforms, incorporating video-logs (vlogs), and collaborating with community influencers, leaders and organizations are essential strategies (Arnold et al., 2022; Hill et al., 2023, 2025a). Additionally, sharing and respecting the stories of Black women already taking PrEP can create a more supportive and empathetic counseling environment (Arnold et al., 2022). This approach can help build trust, allowing women to feel understood and respected, and thus more likely to engage with PrEP. Including Black women in the development and implementation of PrEP programs also ensure their specific concerns and needs are included and considered (Arnold et al., 2022).

Many Black women expressed that PrEP advertisements or promotions do not resonate with their experiences or seem irrelevant to them (Arnold et al., 2022; Troutman et al., 2021; Ayangeakaa et al., 2023). To address this, it is essential to diversify PrEP advertising to be more inclusive of Black women. Utilizing culturally relevant imagery, language, and messaging can make outreach efforts more effective and resonate better with the target audience. Furthermore, Black women mentioned that their healthcare providers often lack knowledge about PrEP and rarely offered PrEP or discuss HIV risk behaviors, such as sexual practices and substance use (Arnold et al., 2022; Shull et al., 2020; Hill et al., 2025c). Several women also indicated that they would have considered PrEP if their healthcare provider had recommended it (Arnold et al., 2022; Storholm et al., 2022). This highlights the crucial role that healthcare providers play in initiating conversations about PrEP and ensuring that patients are informed about its benefits and availability (Hill et al., 2025b).

Medical mistrust rooted in historical and ongoing systemic racism may influence how Black women receive PrEP-related information and their willingness to initiate PrEP. Tekeste et al. (2018) found that while Black women expressed greater interest in learning about PrEP and stronger intention to initiate it if offered at no cost, the study also reported significantly higher levels of medical mistrust compared to White women. Notably, medical mistrust was negatively associated with comfort in discussing PrEP with healthcare providers. These findings underscore the need for culturally sensitive approaches that build trust and strengthen provider-patient communication to address misconceptions, enhance awareness, reduce stigma, and support informed decision-making about sensitive topics such as HIV risk and PrEP prevention (Shull et al., 2020; Hill et al., 2025b,c).

In our pilot study, we utilized two distinct vlogs tailored separately for Black women and healthcare providers as a new intervention strategy to address gaps in PrEP awareness and uptake. Engaging both groups is essential to increase access to PrEP among Black women. While patients may lack awareness or hold misconceptions about PrEP, healthcare providers are in a key position to offer guidance, education, and referrals (Hill et al., 2025b,c). By integrating these efforts, our study aimed to improve PrEP awareness and uptake among Black women.

## Utilizing the emergency department for PrEP initiation

The emergency department is a feasible setting for offering and initiating PrEP due to its high patient volumes, prevalence of non-emergent cases and direct reach to underserved, hard-to-reach populations (Crooks et al., 2023; Irie et al., 2023; Shull et al., 2020). By incorporating PrEP referrals/prescriptions into ED visits, healthcare providers can identify individuals who could benefit from PrEP while they are already in care, eliminating the need for a separate appointment (Jackson et al., 2023; Hill et al., 2025c). For instance, this practical approach is supported by a pilot study by Zhao et al. (2021), which found that most ED patients are interested in prevention education (73%) and open to PrEP referrals (32.8%), with many successfully contacted by peer navigators (74.4%). Similarly, Solnick et al. (2025) reported that participants viewed the ED as a practical setting for PrEP education, especially during wait times, and emphasized its potential to reduce stigma by normalizing these discussions.

Building on the ED's potential as feasible setting, our pilot study leveraged the ED to effectively identify PrEP-eligible Black women and engage healthcare providers in PrEP education and referral. As a part of this effort, tablet devices were used to present vlogs containing important PrEP information to raise awareness, improve PrEP uptake, and facilitate effective linkage to services. However, to ensure the feasibility of this approach, practical implementation challenges must be addressed, including time constraints, competing care priorities, and the limited capacity of ED physicians (Bisom-Rapp et al., 2024). These challenges are also highlighted by Devlin et al. (2024), who reported that although ED physicians and patient advocates viewed the ED as “the perfect place to introduce” PrEP, many physicians noted that limited time often prevents PrEP discussions.

One effective strategy to address these challenges is fostering interdisciplinary collaboration among physicians, nurses, pharmacists, social workers, and patient navigators. This collaborative approach helps distribute the workload, ensuring that patient education and referrals are not the sole responsibility of any one clinician. Supporting this, a study by Bisom-Rapp et al. (2024) involving 22 multidisciplinary clinicians in an ED-based PrEP program found that shared responsibilities across roles reduced individual burden and improved the feasibility of PrEP delivery. Additionally, Yankam et al. (2023) highlight task shifting and task sharing as effective strategies to address workforce shortages in sub-Saharan Africa, underscoring the importance of interdisciplinary collaboration among health professionals. Their insights reinforce the value of team-based approaches to strengthen health system capacity and service delivery.

Another key strategy is integrating electronic health record (EHR) tools to identify at-risk individuals and streamline the PrEP delivery (Devlin et al., 2024). For example, Ridgway et al. (2018) demonstrated that an EMR-based risk tool in the ED resulted in high interest in PrEP (68.6%), scheduling follow-ups (17.6%), and initiating PrEP (7.8%). This study also reported that among cisgender women who had HIV risk assessment and counseling, 61% expressed interested in PrEP. These EHR tools can reduce staff workload and improve ED efficiency by automating lab orders, prescriptions, and patient education topics (Bisom-Rapp et al., 2024). Similarly, Galbraith et al. (2016) used an EHR system for HIV screening and automatic test orders, along with counseling and referrals, which led 76% of diagnosed patients to attend their first clinic visit. In their study, behavioral risk assessments were conducted post-ED visit via phone using CDC PrEP guidelines to identify individuals at substantial risk. A logistic regression model was then developed using EMR data available at triage to generate a numeric risk score for each patient. This score was automatically calculated within the Epic EMR system, triggering alerts for HIV prevention counselors when patients exceeded the risk threshold. Such an EHR-based system could be adapted in ED settings to facilitate quicker and more efficient PrEP referrals.

To maximize the effectiveness of ED-based PrEP delivery, it is essential to provide ED staff with regular trainings and resources (Jackson et al., 2023). Such training should focus on recognizing PrEP as a safe and effective HIV prevention option for adults and sexually active populations (Jackson et al., 2023; Hill et al., 2025b,c). Studies show that with proper training, ED clinicians feel more comfortable discussing PrEP and desire information on both its safety and risks (Bisom-Rapp et al., 2024). Other research has identified confusion among providers between PrEP and post-exposure prophylaxis (PEP), highlighting the need for targeted education to help staff confidently identify eligible patients and initiate PrEP conversations (Devlin et al., 2024).

To further ease staff demands and optimize patient education, utilizing digital tools such as tablet devices or kiosks enables patients to engage in self-guided learning during ED wait times, minimizing the need for direct provider involvement (Hill et al., 2022). Studies indicate most ED patients favor technology-based health interventions, with nearly 90% preferring digital information on topics such as HIV and sexually transmitted infections (Ranney et al., 2012). For example, Chernick et al. (2021) found that the tablet-based intervention *Dr. Erica* reported high enrollment (84.4%), follow-up rates (82.9%), and increased plans for future contraceptive use compared to controls (65.9 vs. 30.0%). Furthermore, most ED patients (93.1%) own smartphones and view digital tools favorably, suggesting that the ED is a promising setting for digital health interventions that align with patients' technology habits and preferences (Goldfine et al., 2022).

Additionally, it is important to consider the patient's emotional state during their ED visit, as this can impact their engagement with digital interventions. Designing brief, engaging, and clear content helps maintain patient engagement, especially when staff briefly introduce the tools and offer support. Research indicated that patient-centered, supportive approaches combined with engaging content are key to successful digital health programs (Rutland et al., 2024; Mindu et al., 2023). Moreover, to overcome infrastructure challenges, EDs should have sufficient devices, technical support, reliable internet connectivity, places to charge devices, and clear privacy rules for patient use. Supporting this, Bailey et al. (2017) found that both staff and patients identified technical difficulties, limited access to IT resources as major barriers to the effective delivery of digital sexual health interventions. Addressing these issues enables smooth integration of digital tools into the ED workflow, improving patient education and PrEP uptake.

## Conclusion

Integrating public health research within an ED setting through eHealth interventions, such as vlogs, offers a promising strategy to enhance preventive care. The experience stemmed from the aforementioned pilot study demonstrated the potential of using the ED visit to reach Black women and healthcare providers and enhance their knowledge, understanding and linkage to PrEP. Despite challenges such as establishing trust, ensuring privacy, and managing time constraints, the potential benefits of this approach are promising. By leveraging the unique strengths of ED settings and addressing barriers through tailored strategies, the public health field can improve the health of populations through preventive health services and support broader public health goals. This approach not only offers a valuable opportunity to reach underserved community members but also underscores the need for continuous adaptation and refinement of strategies to enhance PrEP uptake and improve health outcomes for all.
